# ‘Lamina External Graft Overlay’: The Use of Segmented Xenogenic Bone Sheets in the Reconstruction of 3D Bone Defects

**DOI:** 10.3390/medicina61040683

**Published:** 2025-04-08

**Authors:** Roberto Rossi, Fabrizio Bambini, Claudia Dellavia, Dolaji Henin, Lucia Memè

**Affiliations:** 1 Private Practice Genoa, 16121 Genova, Italy; 2 Department of Clinical Sciences and Stomatology (DISCO), Polytechnic University of Marche, 60121 Ancona, Italy; f.bambini@staff.univpm.it; 3 Department of Biomedical, Surgical and Dental Sciences, University of Milan, 20122 Milan, Italy; 4 Dipartimento di Scienze della Vita, della Salute e delle Professioni Sanitarie, Link Campus University, 06012 Città di Castello, Italy; l.meme@unilink.it

**Keywords:** guided bone regeneration, dental implants, xenogenic bone sheets, resorbable membranes

## Abstract

Guided bone regeneration (GBR) has represented a challenge for clinicians in the past 30 years, and the literature has well described many different surgical options such as d-PTFE membranes, titanium grids, or autogenous bone harvested from the posterior mandible. All of the previously mentioned techniques have shown a high rate of complications but, in the last decade, a new membrane made of xenogenic bone was introduced. Most of the publications regarding its application report very few and mild complications. In this article we will suggest a new application using segmented xenogenic bone sheets instead of autogenous bone to correct severe ridge deformity. *Background and Objectives*: Xenogenic bone sheets have been studied extensively over the past decade and have proven effective, with a very low rate of complications when used to reconstruct bone atrophies. The technique presented in this paper aims to reduce morbidity, avoid the need for intra-oral graft harvesting, and minimize both surgical time and post-operative discomfort. *Materials and Methods*: Xenogenic bone sheets of equine origin were used to reconstruct severe 3D bone defects in five patients requiring dental implants. The segmentation of the sheet allowed the operator to rebuild the missing bone walls and achieve optimal anatomy without compromise. Furthermore, using different sizes and thicknesses of the bone sheets allowed safe procedures preventing early exposure of the membranes. CBCT of the defects before and after 8 months of healing were measured with Exocad software to assess the volumetric gain. Histological analysis performed on one site showed integration of the bone lamina and live bone underneath. *Results*: In all five cases evaluated the ridge deformities were successfully corrected and all patients’ implants have functioned for more than two years to date. The average horizontal bone gain in these five cases was 6.18 mm (±1.19 mm) while the vertical gain was 9.70 mm (±2.39 mm). *Conclusions*: This new application of flex cortical sheets simplifies the surgical procedure for both operator and patient, reduces morbidity and post-operative complications, and shows promising signs for resolving complex 3D bone reconstructions.

## 1. Introduction

The concept of guided bone regeneration (GBR) was introduced over twenty years ago, initially based on animal studies [1,2]. The approach involved isolating a bone defect with a barrier membrane placed between the defect and the surrounding soft tissue. This allowed a blood clot to fill the wound area, promoting bone formation [3]. Initially, the GBR technique was typically performed using non-resorbable barriers made of PTFE and e-PTFE [1,3], with or without additional modifications [4,5]. While these membranes were effective in stabilizing the graft and preventing soft tissue cell proliferation, they were prone to post-operative complications, such as membrane exposure [2,6]. As a result, research shifted toward the development of resorbable barriers. Among these, collagen membranes gained popularity due to their biocompatibility, ease of handling, and chemotactic properties for osteoprogenitor cells [7,8,9]. However, a major concern with collagen membranes is their lack of rigidity and relatively short resorption time, which can pose challenges in large ridge reconstructions [10].

As regards the use of biomaterials, there is a vast scientific literature. These are osteoconductive materials that function as a scaffold for the future matrix [11,12,13].

In order to overcome the limitations of non-resorbable membranes and collagen membranes, rigid structures that could scaffold and mimic bone have been considered. Recently, resorbable bone sheets have been developed that, being composed of partially demineralized cortical bone, act both as a scaffold to promote tissue regeneration and as a protective membrane. These bone sheets can be equine- [14,15,16,17] or porcine-derived [18,19,20,21,22].

The presence of a residual layer of hydroxyapatite imparts slight rigidity to the cortical bone sheet, extending its protective function (>4 months) and enhancing its stability compared to collagen membranes. Another advantage of cortical bone sheets is their resorbability; accidental exposure merely accelerates their degradation by collagenases, with no significant impact on bone regeneration [22].

Posterior ridge deformities have been treated in the last 30 years with many different surgical techniques and biomaterials and devices and there are many different opinions regarding the use of autogenous bone harvesting or using a mix of autogenous bone along with a scaffold [1,2,3,6,9,10,11,19,23,24].

In the last decade the use of xenogenic bone substitutes has produced a number of publications proving the efficiency of bone substitutes [25]. The use of cortical bone membrane has proven to be effective even when applied in different clinical situations, from bone dehiescences to the loss of buccal plate to the combined loss of both cortical plates. Different surgical approaches have shown consistent results clinically, histologically, and in terms of stability of the regenerated bone on long-term follow-up [14,15,16,17,21,22,23,25,26,27]. In this paper we will introduce a new application of xenogenic cortical sheets, mimicking the Khoury technique [26] but using fibrine glue in place of osteosyntesys screws and avoiding harvesting from a second surgical site in the mouth of the patient. Having bone sheets of different thicknesses offers the surgeon a quick and safe way to set up the regeneration site.

In the regeneration of complex and large three-dimensional bone defects, there can be both well-vascularized and poorly vascularized regions. The thickness of the barriers used in each region should be carefully considered to address the biological challenges of these large defects. This case report aims to evaluate the potential of a new surgical technique that utilizes trimmed pieces of FCS with varying thicknesses as barriers in complex 3D ridge augmentation procedures.

## 2. Materials and Methods

### 2.1. Biomaterials

The materials used in this case report included a flex cortical sheet (FCS) of equine origin (Osteoxenon^®^, Flex Cortical Sheet, Bioteck SpA, Arcugnano, Italy) and equine bone granules containing preserved collagen (Osteoxenon^®^, Mix Granules, Bioteck SpA, Arcugnano, Italy), mixed in a 1:1 ratio with autologous bone. Both the FCS and equine bone granules are produced through a patented biochemical process involving enzyme treatment (Zymo-Teck^®^ Process, Bioteck SpA, Arcugnano, Italy). This process selectively removes antigenic components at low temperatures while preserving the natural collagen structure and mineral phase of the bone, which enhances physiological interaction with the patient’s osteoclasts and osteoblasts [28]. As a result, the grafts remodel more quickly, with a higher percentage of new bone formation compared to those produced through traditional processes (i.e., thermal treatments) [29,30]. In addition, the FCS undergoes partial demineralization to provide flexibility upon hydration by partially removing hydroxyapatite and exposing the preserved collagen, allowing the sheet to bend when hydrated. It is crucial to note that FCSs are sourced from the long bones of equines, where the bone fibers run parallel. The orientation of these fibers is indicated by a triangular indentation on the side of the FCS, and the sheet must be folded perpendicularly to this indentation to prevent rupture. Flexibility after hydration allows the clinician to adjust the FCS to fit the defect’s size and geometry accurately.

FCSs are available in thicknesses of 0.2 mm, 0.5 mm, and 0.9 mm, suitable for various defect types, from periodontal/peri-implant defects [15] to complex horizontal and vertical augmentations [14,16]. The FCSs are removed from the package, hydrated in sterile solution for 10–15 s, trimmed, and bent to replicate the missing anatomy. The flexibility of the material allows for natural anatomical reconstruction, unlike autogenous bone plates, which are rigid and require pins or screws for stabilization. Instead, FCSs can be fixed using surgical and/or fibrin glue (FG) in addition to osteosynthesis materials.

### 2.2. Preparation of FG

In this case, fibrin glue (FG) (Tisseel, Baxter, Deerfield, IL, USA) was used to stabilize the bone graft beneath the FCS and to secure the barrier in place. Tisseel 2 mL comes in a dual-chamber syringe: one chamber contains clotting agents (human fibrinogen 91 mg/mL and aprotinin 3000 KIU/mL) and the other contains human thrombin (500 IU/mL) and calcium chloride (40 µmo/mL). Tisseel is stored at −20 °C and needs to be removed from the freezer one hour before use. To dilute the thrombin for GBR application, 1 mL from chamber 2 is extracted with a sterile syringe and mixed with 9 mL of bi-distilled water in a sterile glass container. The diluted thrombin is then returned to chamber 2, providing the clinician with more time to prepare the graft and apply FG to fix the FCS in the desired position.

### 2.3. Surgical Technique and ‘Lamina External Graft Overlay’ (L.E.G.O.) Rationale

Three-dimensional bone reconstruction is always a challenge in oral surgery. Many techniques rely on autogenous bone transplantation [26,27], which is considered the gold standard. However, the use of autogenous bone requires the surgeon to work with bone plates that are sharp, rigid, and difficult to contour to match the local anatomy. This type of procedure demands surgical expertise in harvesting, manipulating, and stabilizing hard tissue, often involving multiple osteosynthesis devices that must be removed at a later stage or during implant placement. In complex defects, oral and maxillofacial surgeons may use segmented autogenous bone plates to build boxes from the various segments, but even this segmentation and shaping requires a high level of skill. The introduction of xenogenic bone sheets has greatly simplified the process of 3D bone augmentation. These sterile bone devices eliminate the need for a donor site, making surgery less invasive. The availability of flexible xenogenic bone sheets, which can be hydrated, bent, and adapted to the surgical site, is a significant advancement. FCSs are available in different thicknesses, allowing their use to be tailored to the gingival phenotype in the area of augmentation. Bone defects are often accompanied by soft tissue defects, so having a membrane that matches the thickness of the soft tissue is a considerable improvement in simplifying the procedure. In complex 3D bone reconstructions like the one presented here, FCSs can be segmented and applied in varying thicknesses depending on the location where the cortical bone sheet is required, i.e., using a thicker FCS in the presence of thicker soft tissues and a thinner FCS in the presence of thin soft tissue. The bony architecture is first reconstructed using a mix of 50% autogenous bone harvested from the surgical site or nearby areas, combined with 50% collagenated equine bone graft. This mixture is hydrated with the patient’s blood clot and further stabilized with a few drops of fibrin glue, creating a stable bone graft that enables volumetric restoration of the defect. Once this step is completed, the FCSs are trimmed to fit the geometry of the defect, hydrated in warm sterile saline for a few seconds, and then applied over the grafted area to protect the graft and the reconstructed cortical plate. The thickness of the FCS is determined during grafting, depending on the location and shape of the defect. This is the basis of the ‘lamina external graft overlay’ (L.E.G.O.) technique.

### 2.4. Patient Selection

Five patients aged 26 to 59, three male and two female, who lost one or more teeth resulting in a severe three-dimensional bone defect were included. All patients were in good health, did not take medications, and had a medical history not reporting metabolic diseases or restrictions to GBR and later to implant therapy. All patients signed an informed consent form for the procedure, knowing the material and methods that were going to be used. They also signed a consent form to take biopsies from the sites to evaluate the quality of the bone regenerated.

### 2.5. Histological and Histomorphometric Analysis (Patient N° 3)

For patient N° 3 it was possible to collect a bone biopsy at the moment of implant placement (8 months from the regenerative surgery) for subsequent histological examination. The bone biopsy shown in this paper was collected and immediately stored in 10% buffered formalin at 4 °C until further processing as a non-decalcified bone specimen. The specimen was dehydrated with an increasing alcohol scale infiltrated and embedded in poly-methylmethacrylate resin (Kulzer Technovit 7200 VLC^®^, Bio Optica, Milano, Italy). The bone sample was cut longitudinally with a diamond blade (Micromet Remet^®^, Bologna, Italy) to obtain 2 sections for each block section. The slices were processed to reach 120.00 ± 20.00 µm thickness and subsequently stained with Toluidine Blue/Pyronin G (Sigma-Aldrich^®^, St. Louis, MO, USA). The sections were examined at different magnifications (100×, 200×, 400×) with an optical microscope (Eclipse E600^®^, Nikon, Tokyo, Japan) connected to a digital camera (DXM1200^®^, Nikon, Tokyo, Japan). Slides were further acquired through a high-resolution scanner at 40× magnification (NanoZoomer, Hamamatsu, Tokyo, Japan). Newly formed bone, residual material, and medullary spaces were calculated using a standard stereologic method. This consists in applying a digital counting grid on each histological section analyzed. Tissue below each grid intersection is classified as mineralized matrix and/or osteoid tissue (newly formed bone), medullary spaces, or residual biomaterial. Volumetric percentages are then calculated by running a ratio on the intersection points afferent to each tissue type and the number of total intersections [31,32].

## 3. Surgical Technique and Case Presentation (Patient N° 3)

### 3.1. Clinical History

A single case (patient N° 3) is presented here in order to provide a detailed description of the procedure and of the results obtained by applying the L.E.G.O. technique.

**Figure 1:**
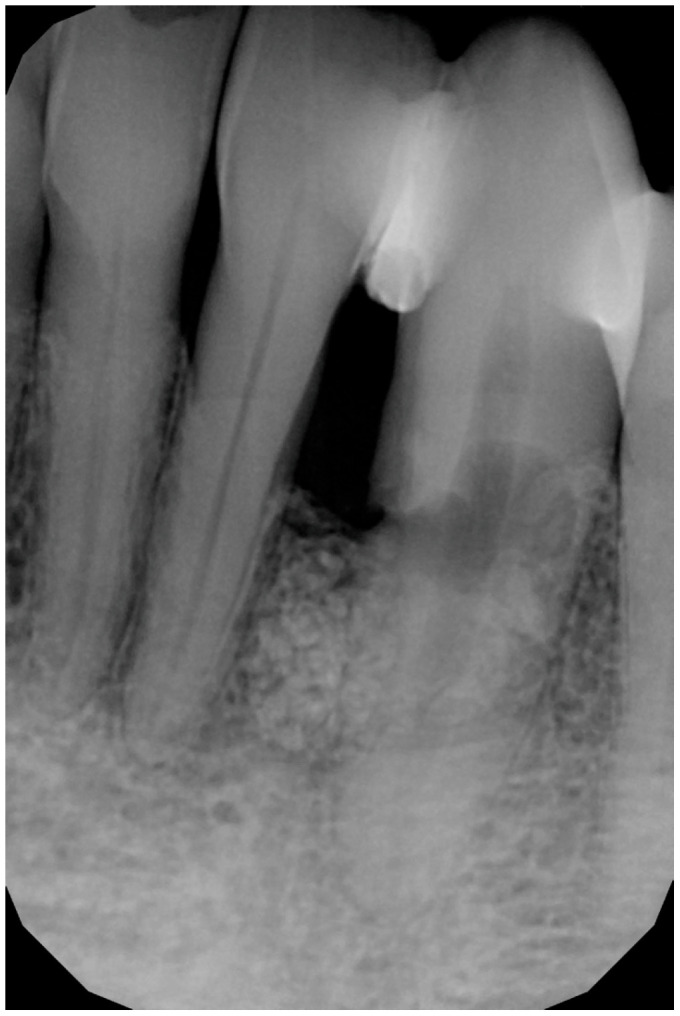
Intraoral X-ray at baseline, highlighting the damaged root of element 33.

A 56-year-old male patient has been under our care for several years. The canine tooth in position 33 was treated 10 years ago using guided tissue regeneration (GTR) to address an infrabony defect. At that time, the biomaterial employed was an anorganic bovine bone-derived matrix (ABBM) in conjunction with a resorbable membrane. After 7 years, the tooth began to exhibit signs of internal root resorption and ultimately fractured (Figure 1).

**Figure 2:**
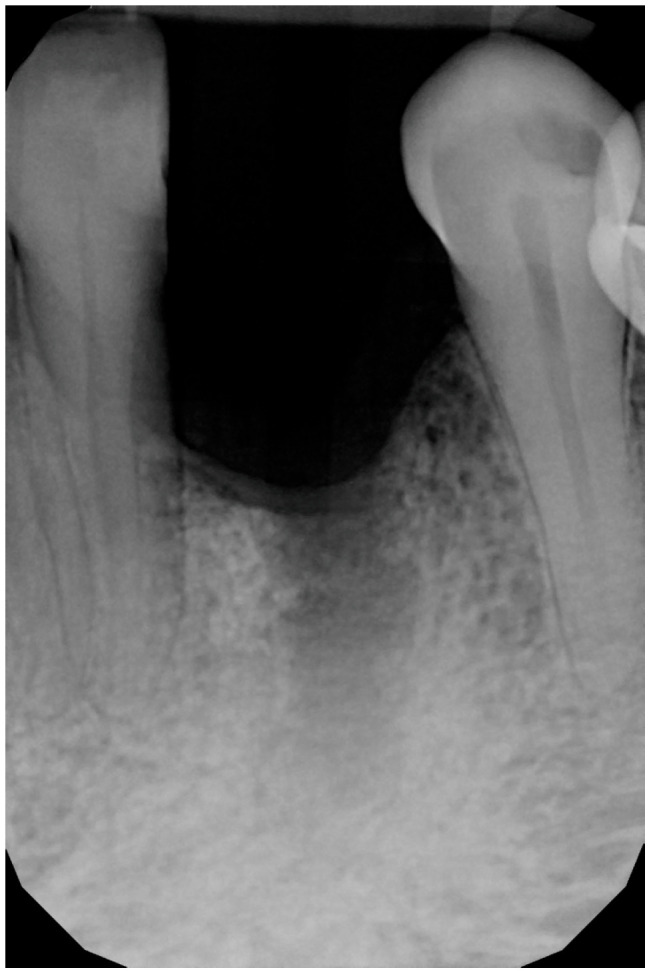
Intraoral radiograph following the extraction of the canine.

**Figure 3:**
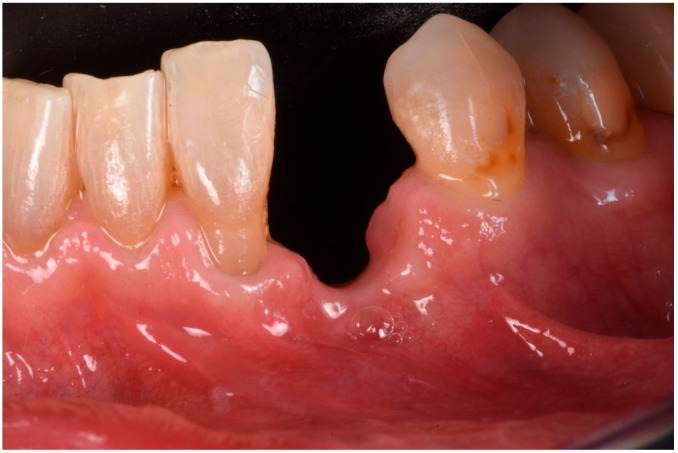
Frontal view of the bone defect three months post-extraction.

**Figure 4:**
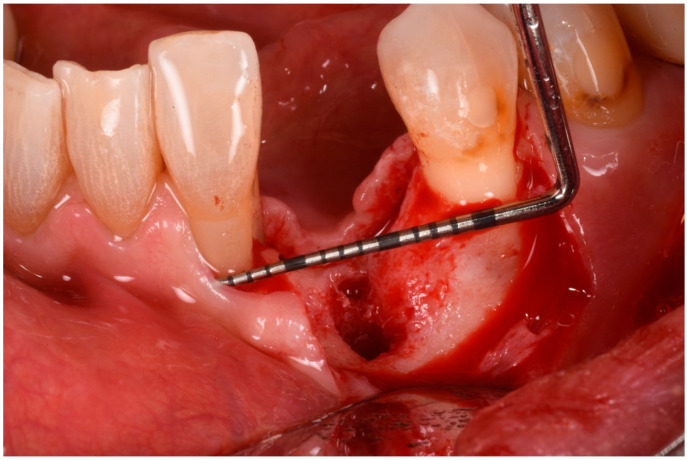
Mesial–distal defect measuring 13 mm, with a significant portion of the buccal plate absent.

**Figure 5:**
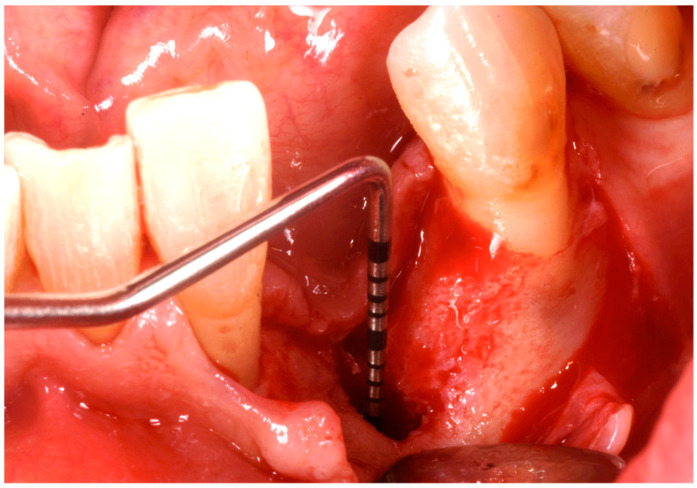
Vertical bone defect measuring 9 mm.

The extraction was performed at another practice while the patient was away for work, and it was conducted on an emergency basis (Figure 2). Three months post-extraction, the patient returned to our clinic, reporting a severe bone defect with significant loss of both the mesial bone peak and the buccal plate (Figure 3, Figure 4 and Figure 5). The proposed plan, which the patient accepted, involved performing a regenerative procedure using an FCS followed by delayed implant rehabilitation.

### 3.2. Hard Tissue Augmentation

**Figure 6:**
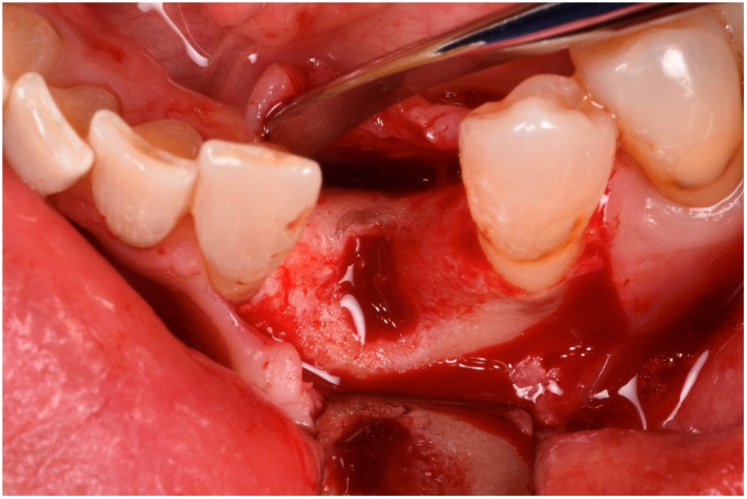
Occlusal view of the bone defect.

The patient was anesthetized with articaine 1:200,000. A flap was designed to extend from the distal aspect of the lateral incisor to the distal of the first bicuspid. In this case, only one releasing incision was made distal to the bicuspid to provide greater flexibility at the mesial aspect, where bone loss was more advanced. A full-thickness buccal and lingual flap was elevated to expose the 3D lesion. The mesial–distal component of the defect measured 13 mm, with a significant portion of the buccal plate completely missing (Figure 4). The vertical component of the defect comprised 9 mm of suprabony component and 4 mm of infrabony component (Figure 5). The horizontal bone loss between the lingual and buccal walls was measured at 8 mm (Figure 6).

**Figure 7:**
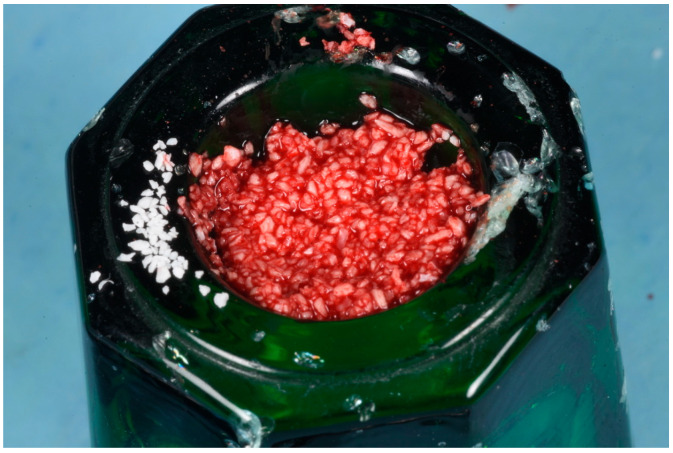
Equine bone granules, maintaining preserved collagen, mixed in a 1:1 ratio with autologous bone.

**Figure 8:**
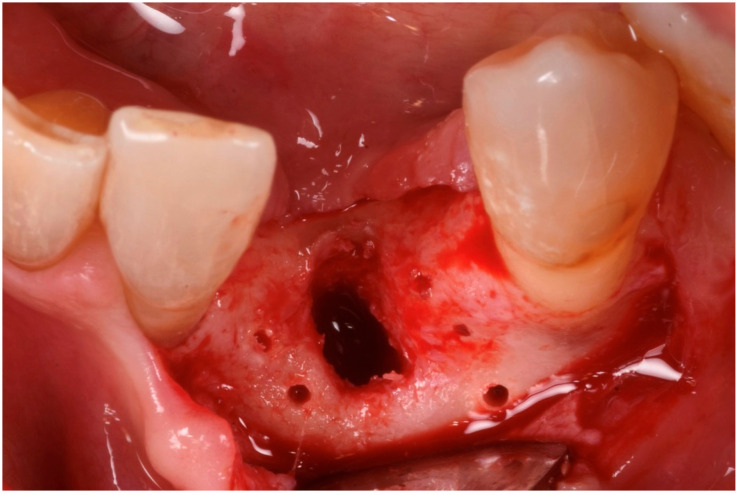
The recipient site prepared to receive the graft.

**Figure 9:**
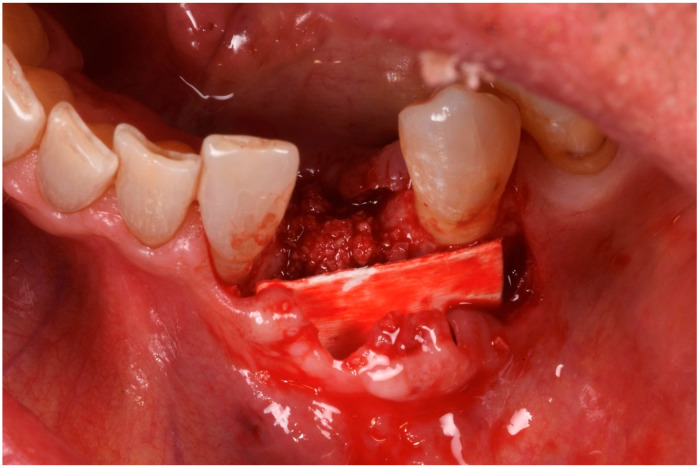
The 0.9 mm thick fibrin sealant (FCS) fixed on the vestibular side.

**Figure 10:**
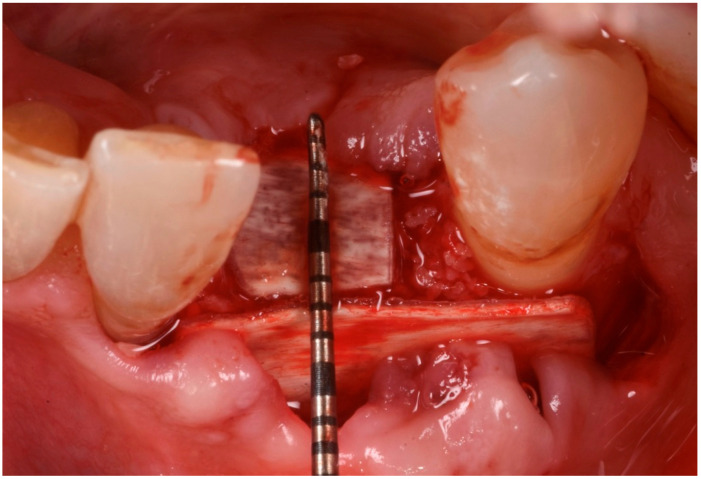
Both FCSs in position.

The blood clot was collected from the wound area and mixed with collagenated equine bone granules and autogenous bone scraped from the lingual side of the defect, along with a few drops of fibrin glue (FG) (Figure 7). This mixture ensured that the graft was both malleable and sticky, allowing it to adhere effectively to the base of the defect. The recipient site was prepared using a small round bur to create intramarrow penetrations, which facilitated additional bleeding and introduced mesenchymal cells from the marrow spaces (Figure 8). In this case, two different thicknesses of FCS were used: the thicker one (0.9 mm) was applied to restore the missing buccal plate, while a thinner FCS (0.5 mm) was utilized to protect the occlusal aspect of the defect and to seal the graft underneath (Figure 9 and Figure 10).

The FCSs were hydrated for a few seconds in sterile saline, trimmed, soaked with the patient’s blood clot, and secured in place by adding FG to the periphery while applying gentle pressure for 15 to 20 s. Once the buccal plate was reconstructed, the bone graft was inserted into the now four-wall defect (Figure 9). The graft was gently compressed to fill the defect completely. The next step involved preparing the lingual and coronal FCSs. Following this procedure, the width of the restored ridge measured approximately 8 mm.

**Figure 11:**
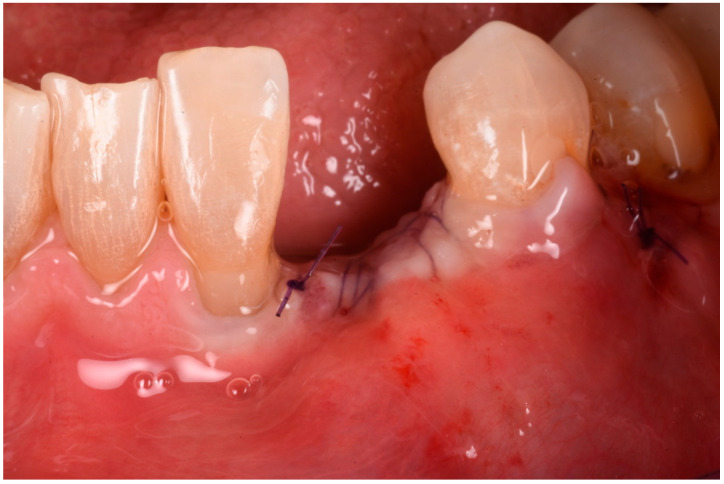
Flaps sutured in place.

**Figure 12:**
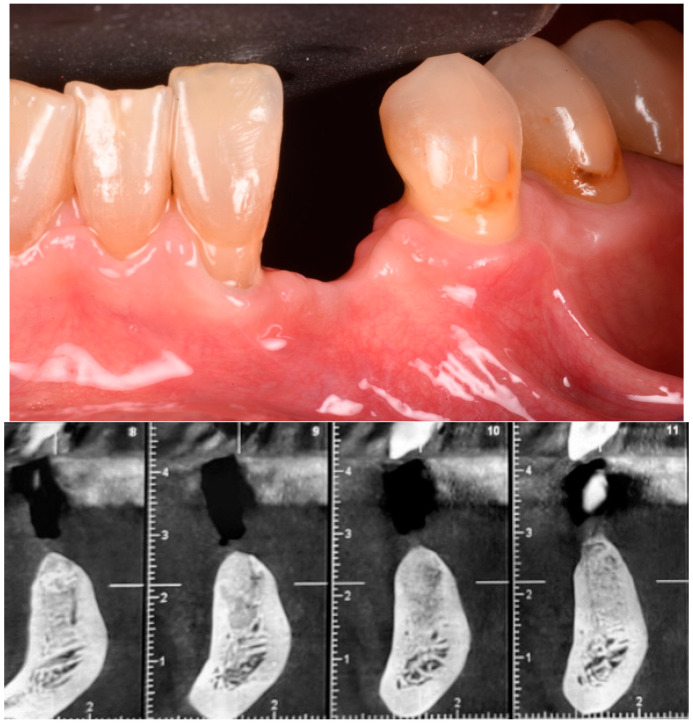
Soft tissue healing 8 months post-op and CBCT proving the shape of the ridge.

The buccal flap was extended from distal to mesial using a technique known as ‘soft brushing’, performed with dedicated atraumatic instruments. This method allows the flaps to be stretched without sharp incisions, which reduces post-operative edema and results in soft tissues that are well adapted to the new anatomy. To ensure a perfect and tight seal of the flap, 5–0 resorbable sutures (two double-sling sutures at the mesial and distal papilla and a horizontal locking mattress in the middle of the ridge) were used (Flysorb Mono Butterfly, Cavenago di Brianza, MB, Italy), protecting the augmented site (Figure 11). The post-operative protocol included amoxicillin 1 g twice daily for a week, ibuprofen 400 mg as needed for discomfort, and gentle rinsing with 0.2% chlorhexidine spray once a day. Eight months post-surgery, healing was uneventful, with soft tissues appearing at the same level as on the surgical day. Concurrently, a CBCT was performed to assess the degree of healing and integration of the xenografts and to plan the insertion of the implant. The CBCT results indicated a good degree of mineralization, suggesting it was time to position the planned implant (Figure 12).

### 3.3. Implant Insertion and Follow-Up

**Figure 13:**
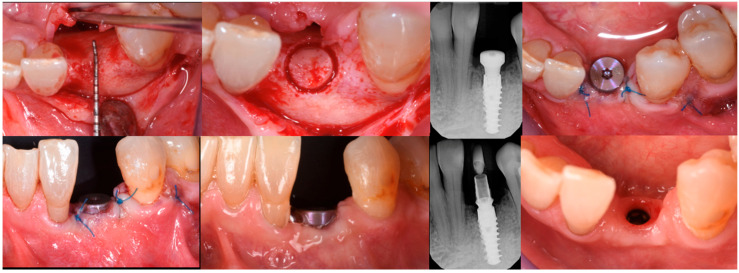
The sequence of biopsy, implant insertion, and healing.

On the day of surgical re-entry for implant insertion, the area was anesthetized with articaine 1:200,000, and full-thickness flaps were elevated to expose the surgical site. Clinically, newly formed bone and the two FCSs that had fully integrated into the local anatomy were observed. A trephine with a 4 mm inner diameter was used to collect a biopsy from the area for histological analysis. A 4.5 mm × 13 mm implant (Institute Straumann, Basel, Switzerland) was placed at the site. Given that the implant stability quotient (ISQ) measured above 70, a healing abutment was also placed, and the soft tissue was sutured around it. Two months post-implant placement, the healing of the soft tissues was uneventful. A temporary crown was then placed to condition the soft tissue at the site (Figure 13).

**Figure 14:**
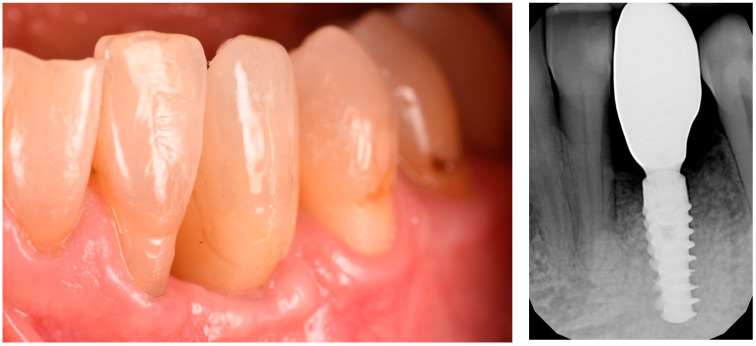
Eighteen-month follow-up after implant rehabilitation, demonstrating optimal maintenance of soft tissues.

**Figure 15:**
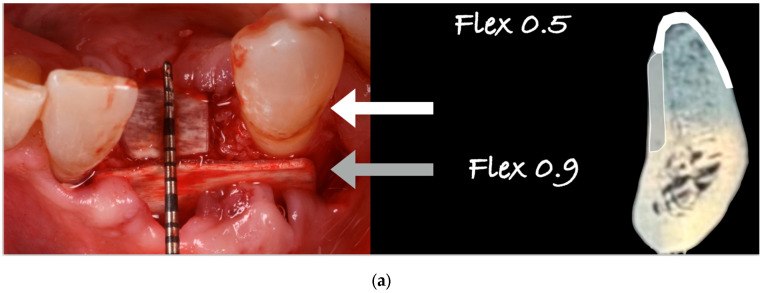

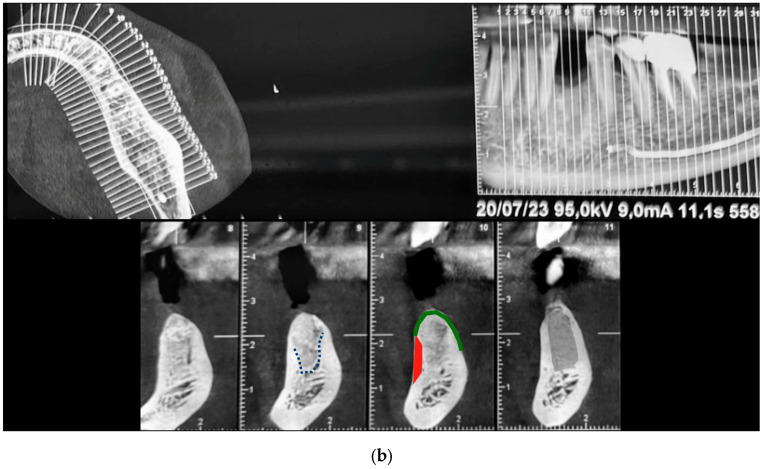
(**a**) Schematic view illustrating the placement of the two FCSs. The 0.5 mm FCS (white) was positioned coronally to protect the bone graft and promote soft tissue healing. The 0.9 mm FCS (light gray) was applied on the buccal side to support the regeneration of the cortical profile and safeguard the bone graft. (**b**) The drawing shows in blue the defect, in green the flex 0.9 and the flex 0.5, and in gray the underlying bone graft.

After three uneventful months, the final impression was taken, and a zirconia restoration was secured in position. The pictures and radiographs taken at the 18-month follow-up showed perfect stability of the soft and hard tissue complex (Figure 14). A summary of the procedure is provided in Figure 15a,b.

### 3.4. Histological Analysis

**Figure 16:**
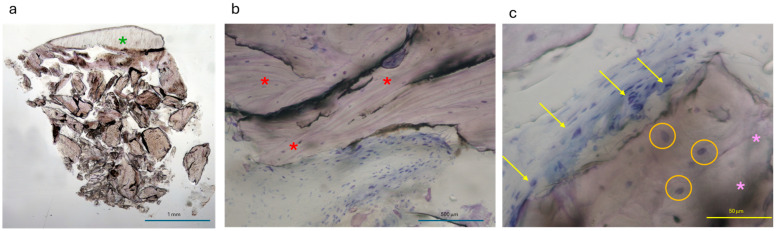
(**a**) Overview of the collected sample. The green asterisk points to the residual cortical bone sheet (FCS). (**b**) A 200× magnification showing different levels of bone mineralization. Red asterisks indicate lamellar bone. (**c**) A 400× magnification showing an active phase of remodeling of collagenated equine bone granules. Pink asterisks indicate residual of biomaterial, yellow arrows highlight active osteoblasts, while orange circles show osteocytes.

The histological evaluation of the bone sample collected during implant placement (8 months post-regenerative procedure) revealed the presence of residual FCS (Figure 16a). This observation indicated that the FCS continued to exert a barrier effect while gradually integrating into the newly formed bone. The histological sections exhibited extensive areas of lamellar bone. Additionally, residual biomaterial was observed, surrounded by osteoblasts actively depositing new osteoid matrix (Figure 16b,c). Histomorphometric analysis demonstrated 36% newly formed bone, 31% residual biomaterial, and 33% medullary spaces. Notably, there were no signs of inflammatory infiltrates or necrotic areas, and neo-angiogenesis was prominently represented.

## 4. Results

**Figure 17:**
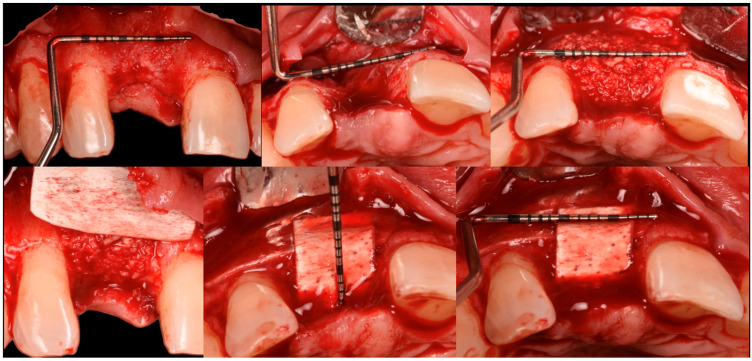
Intraoperatory measurements.

**Figure 18:**
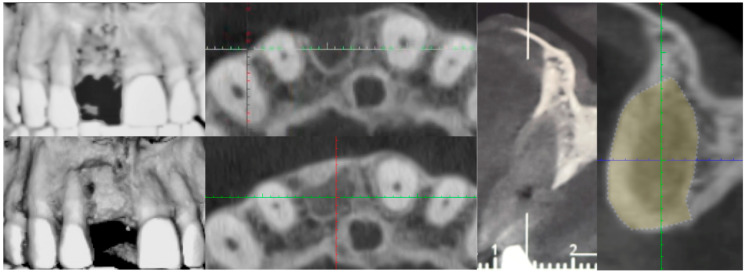
CBCT Comparison before and after augmentation. In yellow the new volume.

**Figure 19:**
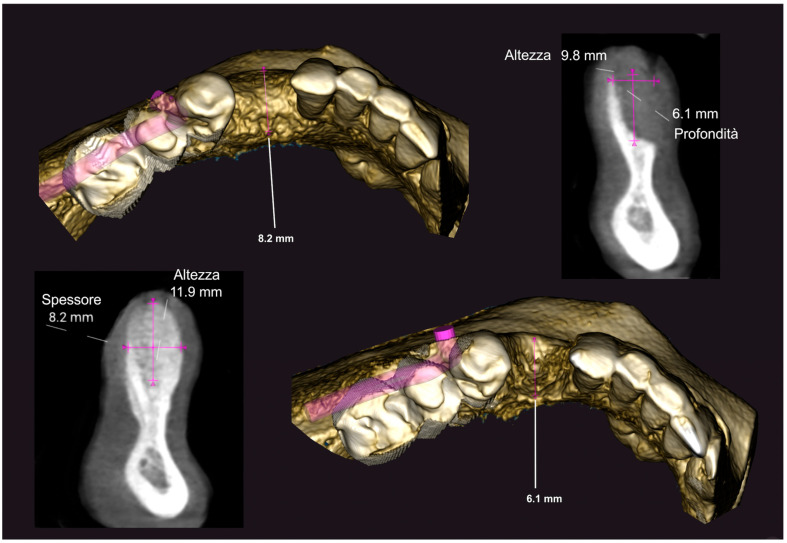
CBCT comparison, baseline and 8 months after GBR.

**Figure 20:**
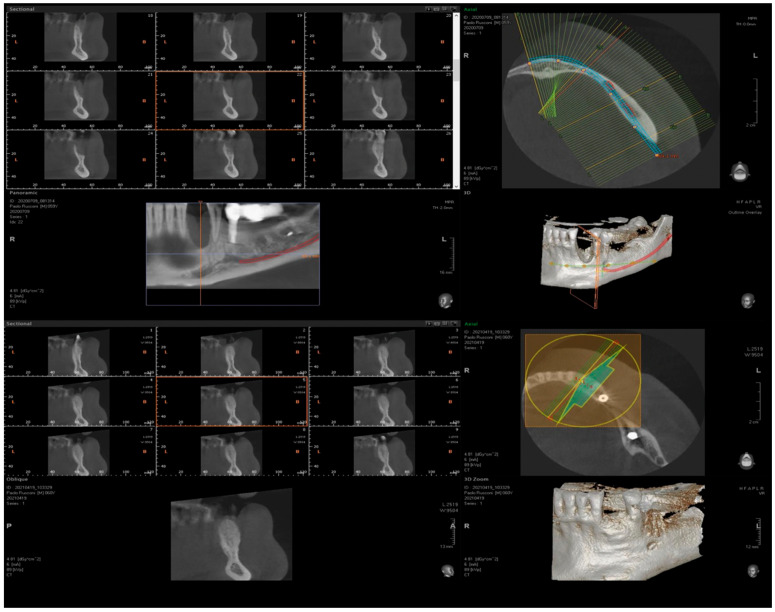
Comparison of CBCT at baseline and 8 months after GBR.

Five sites in five patients of ages 26 to 59 with severe ridge deformities were treated with segmented bone lamina. In Table 1 we report the position and extent of the defects. All lesions were measured by CBCT and at surgery with a 15 mm PCP periodontal probe (Figure 17). The thickness of the residual buccal or lingual wall was measured and recorded and as a vertical component the reference point of measurement was the highest bone peak available. After the bone graft and the bone lamina were glued in position the site was re-measured to calculate the augmentation at baseline. Eight months after augmentation the pre-operative CBCT and the follow up CBCT were compared to re-measure the outcome of the procedure (Figure 18). The volumetric changes were evaluated by comparison using Exocad software and on the CBCT on the cross-sections where augmentation was performed (Figure 19 and Figure 20).

In this small sample, four of the residual bone defects had at least one wall of bone available while only one case had both buccal and lingual walls completely missing. In this case the reference point used to assess measurements was the highest bone peak available at the adjacent tooth. The average gain in the horizontal component was 6.18 mm (±1.19 mm), while the average vertical augmentation was 9.70 mm (±2.39 mm).

## 5. Discussion

Guided bone regeneration (GBR) procedures were developed and introduced after 1988 [31]. Over the years, the technique has been refined to reduce invasiveness (by minimizing autologous bone harvesting) and increase ease of application, alongside continuous advancements in materials and methods within the field of dentistry [32,33]. The original technique employed non-resorbable (PTFE) membranes, which required removal at a second stage during implant insertion, often presenting complications and in a recent study also insignificant new bone formation [33,34,35,36]. The evolution of biomaterials led to the introduction of resorbable membranes, aimed at reducing surgical steps and avoiding post-operative complications associated with non-resorbable membranes, such as early exposure [33]. However, collagen membranes presented limitations, particularly their fast resorption and lack of rigidity, which are essential for stabilizing and protecting bone grafts in complex three-dimensional reconstructions. In the last decade, a new type of barrier made from cortical bone has emerged as a viable solution. Cortical bone sheets, which are resorbable barriers made from xenogenic bone, offer flexibility and adaptability to various defects. Numerous publications and case reports have demonstrated the efficacy of these devices, with long-term follow-ups indicating their predictability and stability over time [12,13,14,15,16,17,18,19,20,21,22,23]. The cases presented in this report aimed to evaluate the use of such barriers in different thicknesses and placements within a complex 3D defect, achieving success from both clinical and aesthetic perspectives. The surgical time was minimal (under 50 min), healing was uneventful, and patient feedback was overwhelmingly positive. The ability to utilize a biomaterial of bone origin represents a significant advancement, alleviating the trauma associated with autogenous bone harvesting. Modern collagenated biomaterials have been shown in numerous studies to be a valid alternative to autografts [12,14,29,30,34,35,36]. In this case series, the sites were restored with substantial bone volumes in all three dimensions, utilizing a novel application of flex cortical sheets referred to as ‘lamina external graft overlay’ (which assembles multiple pieces of trimmed flex cortical bone sheets). The segmentation of the FCS allows clinicians to tailor the number of pieces required based on the specific features of the defect. The grafting material can consist of a combination of autogenous bone harvested from the grafting site and a bone graft, or a bone graft alone, hydrated with the patient’s blood clot and FCS. The flexibility of the FCS, adhered to the underlying bone, enables clinicians to recreate a natural anatomical contour without the need for pins or screws (Figure 17). In the case featured in this paper, a 0.9 mm FCS was utilized to reconstruct the missing buccal plate and protect the graft, while a 0.5 mm FCS was employed to safeguard the graft on the occlusal and lingual sides. The thinner FCS was positioned on the occlusal side to facilitate blood flow from the grafted area to the overlying soft tissue, whereas the 0.9 mm FCS provided greater rigidity, suitable for vestibular wall reconstruction, well supported by a broader buccal flap.

An FCS serves as a semi-permeable membrane, becoming saturated with the patient’s blood clot, which enhances revascularization of the soft tissue covering both the FCS and graft. Machine-made equine cortical bone sheets offer reproducible and precise thickness, which is critical for performing ridge augmentation and GBR of peri-implant and periodontal defects [12,13,14,15,16]. The two FCSs utilized in this case provided effective protection to the grafted defect and contributed to the formation of new buccal and coronal plates (Figure 18). Healing times corresponded with the severity of the initial bone defect, and while the body may take time to ‘digest’ and restructure the xenograft, the end result is viable bone, as evidenced by the histological sections. The integration of equine bone granules into the newly formed bone was substantial, with 36% newly formed bone recorded. The histological analysis also indicated that the 0.5 mm FCS remained visible, suggesting its potential as a long-term membrane. Further studies will be necessary to confirm the positive outcomes associated with the L.E.G.O. technique seen in this small sample. However, the positive results seen in this preliminary investigation highlight the need for clinicians to explore this innovative approach. Large defects can be addressed using a segmented technique, with the option of planning through STL files of patient CBCT scans, simulating the surgical procedure, and determining the precise size and shape of the bony segments, thus simplifying the process and reducing surgical time. In less complex or extensive defects, combinations of FCSs of varying thicknesses, such as 0.5 and 0.2 mm, or solely 0.5 mm, may also be viable alternatives. The optimal combination of FCSs should be determined on a case-by-case basis, tailored to the unique anatomical conditions of the defect being treated. A further benefit of the use of FCSs in clinical conditions as those shown in this case is that, in the unfortunate event of mishandling or mispositioning of FCSs or the flaps, once a lamina becomes exposed, it hydrolyzes and soft tissue heals spontaneously by second intention without severely compromising the regeneration [19]. Cortical bone membranes represent a true innovation because they maintain their nature, flexibility, and rigidity but behave when exposed as ‘resorbable’ membranes, thus limiting the rate of complications and rarely affecting the final outcome of the procedure [12,13,14,15,16,17,18,19,20,21,22,23].

## 6. Conclusions

The technique introduced in this manuscript is straightforward and predictable. A primary goal of modern GBR is to enable planning and execution of bone augmentation in a simple, rapid, and reliable manner. Reducing the need for a second surgical site minimizes the risks of post-operative pain and complications, likely increasing patient willingness to undergo less invasive procedures. Additionally, the excellent cost–benefit ratio associated with this approach is a critical factor in contemporary therapeutic treatment planning. The positive outcomes and favorable patient feedback should encourage future prospective studies involving larger patient populations.

## Figures and Tables

**Table 1:** Values of horizontal and vertical bone augmentation following the procedure.

	Horizontal Bone Width (mm)			Vertical Bone (mm)	
	Baseline	At 8 Months	Augmentation Obtained	Defect Depth	Augmentation Obtained at 8 Months
Patient #1	1.2	8.3	7.1	−9.8	11.9
Patient #2	2	7.8	5.8	−11.2	12.5
Patient #3	1.5	8	6.5	−9.3	9
Patient #4	0	7.2	7.2	−6.8	8
Patient #5	2.5	6.8	4.3	−6	7.1

## Data Availability

The data presented in this study are available on request from the corresponding author upon reasonable request.

## References

[1] Hammerle C.H., Jung R.E. (2003). Bone augmentation by means of barrier membranes. Periodontol. 2000.

[2] Becker W., Hujoel P., Becker B.E. (2002). Effect of barrier membranes and autologous bone grafts on ridge width preservation around implants. Clin. Implant Dent. Relat. Res..

[3] Rocchietta I., Fontana F., Simion M. (2008). Clinical outcomes of vertical bone augmentation to enable dental implant placement: A systematic review. J. Clin. Periodontol..

[4] Urban I.A., Jovanovic S.A., Lozada J.L. (2009). Vertical ridge augmentation using guided bone regeneration (GBR) in three clinical scenarios prior to implant placement: A retrospective study of 35 patients 12 to 72 months after loading. Int. J. Oral Maxillofac. Implant..

[5] Wagner-Ecker M., Voltz P., Egermann M., Richter W. (2013). The collagen component of biological bone graft substitutes promotes ectopic bone formation by human mesenchymal stem cells. Acta Biomater..

[6] Liu G., Hu Y.Y., Zhao J.N., Wu S.J., Xiong Z., Lu R. (2004). Effect of type I collagen on the adhesion, proliferation, and osteoblastic gene expression of bone marrow-derived mesenchymal stem cells. Chin. J. Traumatol..

[7] Regazzoni C., Winterhalter K.H., Rohrer L. (2001). Type I collagen induces expression of bone morphogenetic protein receptor type II. Biochem. Biophys. Res. Commun..

[8] Sbricoli L., Guazzo R., Annunziata M., Gobbato L., Bressan E., Nastri L. (2020). Selection of Collagen Membranes for Bone Regeneration: A Literature Review. Materials.

[9] Banyard D.A., Bourgeois J.M., Widgerow A.D., Evans G.R. (2015). Regenerative biomaterials: A review. Plast. Reconstr. Surg..

[10] Bose S., Roy M., Bandyopadhyay A. (2012). Recent advances in bone tissue engineering scaffolds. Trends Biotechnol..

[11] Kao S.T., Scott D.D. (2007). A review of bone substitutes. Oral Maxillofac. Surg. Clin. N. Am..

[12] Rossi R., Memè L., Strappa E.M., Bambini F. (2023). Restoration of Severe Bone and Soft Tissue Atrophy by Means of a Xenogenic Bone Sheet (Flex Cortical Sheet): A Case Report. Appl. Sci..

[13] Di Stefano D.A., Piattelli A., Zaniol T., Iezzi G. (2020). Implant and Prosthetic Success Following Peri-Implant Guided Bone Regeneration in the Esthetic Zone Using an Equine Cortical Bone Membrane and an Equine Enzyme-Treated Bone Graft: A Retrospective Study with 9-Year Follow-Up. Int. J. Oral Maxillofac. Implant..

[14] Di Stefano D.A., Garagiola U., Andreasi Bassi M. (2017). Preserving the Bone Profile in Anterior Maxilla Using an Equine Cortical Bone Membrane and an Equine Enzyme-Treated Bone Graft: A Case Report with 5-Year Follow-Up. J. Contemp. Dent. Pract..

[15] Di Stefano D.A., Cazzaniga A., Andreasi Bassi M., Ludovichetti M., Ammirabile G., Celletti R. (2013). The use of cortical heterologous sheets for sinus lift bone grafting: A modification of Tulasne’s technique with 7-year follow-up. Int. J. Immunopathol. Pharmacol..

[16] Foti V., Savio D., Rossi R. (2021). One-Time Cortical Lamina: A New Technique for Horizontal Ridge Augmentation. A Case Series. Br. J. Healthc. Med. Res..

[17] Rossi R., Ghezzi C., Tomecek M. (2020). Cortical lamina: A new device for the treatment of moderate and severe tridimensional bone and soft tissue defects. Int. J. Esthet. Dent..

[18] Rossi R., Foce E. (2019). Reconstruction of a horizontal and vertical bone defect using the Cortical Lamina Technique. Arch. Med. Res..

[19] Lopez M.A., Andreasi Bassi M., Confalone L., Carinci F., Ormianer Z., Lauritano D. (2016). The use of resorbable cortical lamina and micronized collagenated bone in the regeneration of atrophic crestal ridges: A surgical technique. Case series. J. Biol. Regul. Homeost. Agents.

[20] Schuh P.L., Bolz W., Weiss M., Niedermaier R., Riemann M., Stelzle F., Wachtel H. (2015). The multi layer technique: An innovative technique of immediate implant placement with simultaneous hard and soft tissue augmentation in the esthetic zone. Implantologie.

[21] Schuh P.L., Wachtel H., Beuer F., Goker F., Del Fabbro M., Francetti L., Testori T. (2021). Multi-Layer Technique (MLT) with Porcine Collagenated Cortical Bone Lamina for Bone Regeneration Procedures and Immediate Post-Extraction Implantation in the Esthetic Area: A Retrospective Case Series with a Mean Follow-Up of 5 Years. Materials.

[22] Rossi R., Foce E., Scolavino S. (2017). The Cortical Lamina Technique: A new option for alveolare ridge augmentation, procedure, protocol and case report. J. Leban. Dent. Assoc..

[23] Rossi R., Barone A., Choukroun J., D’Amato S., Fairbairn P.J., Foce E., Fontana F., Foti V., Gluckman H., Grassi A., Rossi R. (2024). Building Better Bone. A Comprehensive Guide to GBR Techniques.

[24] Perrotti V., Nicholls B.M., Piattelli A. (2009). Human osteoclast formation and activity on an equine spongy bone substitute. Clin. Oral Implant. Res..

[25] Di Stefano D.A., Zaniol T., Cinci L., Pieri L. (2019). Chemical, Clinical and Histomorphometric Comparison Between Equine Bone Manufactured Through Enzymatic Antigen-Elimination and Bovine Bone Made Non-Antigenic Using a High-Temperature Process in Post-Extractive Socket Grafting. A Comparative Retrospective Clinical Study. Dent. J..

[26] Khoury F., Hanser T. (2019). Three-Dimensional Vertical Alveolar Ridge Augmentation in the Posterior Maxilla: A 10-Year Clinical Study. Int. J. Oral Maxillofac. Implant..

[27] De Stavola L., Tunkel J. (2013). Results of vertical bone augmentation with autogenous bone block grafts and the tunnel technique: A clinical prospective study of 10 consecutively treated patients. Int. J. Periodontics Restor. Dent..

[28] Di Stefano D.A., Gastaldi G., Vinci R., Cinci L., Pieri L., Gherlone E. (2015). Histomorphometric comparison of enzyme-deantigenic equine bone and anorganic bovine bone in sinus augmentation: A randomized clinical trial with 3-year follow-up. Int. J. Oral Maxillofac. Implant..

[29] Canullo L., Wiel Marin G., Tallarico M., Canciani E., Musto F., Dellavia C. (2016). Histological and Histomorphometrical Evaluation of Postextractive Sites Grafted with Mg-Enriched Nano-Hydroxyapatite: A Randomized Controlled Trial Comparing 4 Versus 12 Months of Healing. Clin. Implant Dent. Relat. Res..

[30] Carmagnola D., Pispero A., Pellegrini G., Sutera S., Henin D., Lodi G., Achilli A., Dellavia C. (2024). Maxillary sinus lift augmentation: A randomized clinical trial with histological data comparing deproteinized bovine bone grafting vs. graftless procedure with a 5–12-year follow-up. Clin. Implant Dent. Relat. Res..

[31] Dahlin C., Linde A., Gottlow J., Nyman S. (1988). Healing of bone defects by guided tissue regeneration. Plast. Reconstr. Surg..

[32] Elgali I., Omar O., Dahlin C., Thomsen P. (2017). Guided bone regeneration: Materials and biological mechanisms revisited. Eur. J. Oral Sci..

[33] Fontana F., Maschera E., Rocchietta I., Simion M. (2011). Clinical classification of complications in guided bone regeneration procedures by means of a nonresorbable membrane. Int. J. Periodontics Restor. Dent..

[34] Marian D., Toro G., D’Amico G., Trotta M.C., D’Amico M., Petre A., Lile I., Hermenean A., Fratila A. (2024). Challenges and Innovations in Alveolar Bone Regeneration: A Narrative Review on Materials, Techniques, Clinical Outcomes, and Future Directions. Medicina.

[35] Raabe C., Cafferata E.A., Couso-Queiruga E., Chappuis V., Ramanauskaite A., Schwarz F. (2025). Impact of Two Flap Advancement Techniques and Periosteal Suturing on Graft Displacement During Guided Bone Regeneration. Clin. Implant. Dent. Relat. Res..

[36] Alarcón-Apablaza J., Godoy-Sánchez K., Jarpa-Parra M., Garrido-Miranda K., Fuentes R. (2024). Tissue Sources Influence the Morphological and Morphometric Characteristics of Collagen Membranes for Guided Bone Regeneration. Polymers.

